# The secreted acid trehalase encoded by the *CgATH1* gene is involved in *Candida glabrata* virulence

**DOI:** 10.1590/0074-02760200401

**Published:** 2020-10-30

**Authors:** Rafael G Lopes, Julián E Muñoz, Ludmila M Barros, Sergio L Alves-Jr, Carlos P Taborda, Boris U Stambuk

**Affiliations:** 1 Universidade Federal de Santa Catarina, Centro de Ciências Biológicas, Departamento de Bioquímica, Florianópolis, SC, Brasil; 2 Universidade de São Paulo, Instituto de Ciências Biomédicas, Departamento de Microbiologia, São Paulo, SP, Brasil; 3 Universidade de São Paulo, Faculdade de Medicina, Instituto de Medicina Tropical de São Paulo, Departamento de Dermatologia, Laboratório de Micologia Médica/LIM53, São Paulo, SP, Brasil; 4 Universidad del Rosario, Escuela de Medicina y Ciencias de la Salud, Bogotá, Colombia; 5 Universidade Federal da Fronteira Sul, Laboratório de Bioquímica e Genética, Chapecó, SC, Brasil

**Keywords:** ATH1, acid trehalase, neutral trehalase, NTH1, NTH2, extracellular trehalose assimilation, virulence, pathogenic yeasts

## Abstract

**BACKGROUND:**

*Candida glabrata* yeast is the second cause of candidiasis worldwide. Differs from other yeasts since assimilates only glucose and trehalose (a characteristic used in rapid identification tests for this pathogen) by secreting into the medium a highly active acid trehalase encoded by the *CgATH1* gene.

**OBJECTIVE:**

This study aimed to characterise the function of the acid trehalase in the physiopathology of *C. glabrata*.

**METHODS:**

Gene deletion was performed to obtain a mutant *ath1*Δ strain, and the ability of the *ath1*Δ strain to grow in trehalase, or the presence of trehalase activity in the *ath1*Δ yeast cells, was verified. We also tested the virulence of the *ath1*Δ strain in a murine model of infection.

**FINDINGS:**

The *ath1*Δ mutant strain grows normally in the presence of glucose, but loses its ability to grow in trehalose. Due to the high acid trehalase activity present in wild-type cells, the cytoplasmic neutral trehalase activity is only detected in the *ath1*Δ strain. We also observed a significantly lower virulence of the *ath1*Δ strain in a murine model of infection with either normal or immunocompromised mice.

**MAIN CONCLUSIONS:**

The acid trehalase is involved in the hydrolysis of external trehalose by *C. glabrata*, and the enzyme also plays a major virulence role during infectivity.

Fungi are recognised as major agents of nosocomial infections, with emphasis on *Candida* spp. responsible for the majority of invasive fungal infections. Immunocompromised individuals [due to acquired immune deficiency syndrome (AIDS), diabetes, organ and tissue transplantation, cancer treatments, immune-related diseases, premature birth or advanced age] are at high risk for mucosal or systemic candidiasis. Disseminated candidiasis is a life-threatening disease that remains the most common bloodstream infection in hospitalised patients worldwide. Although the majority of fungal infections in humans are caused by *Candida albicans*, an increasing number of cases are being attributed to other *Candida* species, in particular *Candida glabrata*.^1^ Over the last decades *C. glabrata* has emerged as one of the most common and problematic causes of invasive candidiasis, even in Brazil.^2^ Systemic infections caused by this yeast have high mortality rates and are difficult to treat due to this yeast’s frequent and intrinsic antifungal resistance.^3^ This opportunistic pathogen, formerly known as *Torulopsis glabrata*, can be found as a commensal yeast in healthy individuals, and in the environment mainly in association with mammals, reflecting its commensal lifestyle. Indeed, in some populations (such as diabetics and elderly) *C. glabrata* may be the dominant fungal pathogen.

*C. glabrata* and *C. albicans* are quite distinct phylogenetically, suggesting that association of these yeasts with the mammalian host evolved independently in these two pathogens.^4^ *C. glabrata* is closely related to *Saccharomyces* yeasts, and part of the *Saccharomycetaceae* genus named *Nakaseomyces* that contains few other pathogenic species.^5^ During their evolution, these yeasts underwent a whole-genome duplication (WGD) event, and while the genome of most *Saccharomyces* yeasts retained or even expanded some gene families to allow, for example, efficient sugar utilisation and fermentation,^6^ the *C. glabrata* genome underwent a reductive evolution to streamline its metabolic capacity for its life and success as a commensal pathogen.^4^ ^,^ ^5^ For example, the loss of the whole pathway to synthesise nicotinic acid, a precursor of NAD^+^, made this yeast dependent on an external source (e.g., the host) to obtain this vitamin. During the urinary tract infection, the limitation of nicotinic acid is an inducing signal for the expression of the *EPA* genes, a family of adhesins encoded at subtelomeric *loci* subject to transcriptional silencing mediated by the NAD^+^-dependent histone deacetylase *SIR2*.^7^ The auxotrophy of *C. glabrata* for other vitamins (e.g., thiamin and pyridoxine^8^) has also been used for the biotechnological production of pyruvic acid, and other organic acids, from glucose fermentation by this yeast.

Indeed, as *S. cerevisiae* and other members of the *Saccharomycetaceae* clade, *C. glabrata* is a Crabtree-positive yeast that efficiently ferments glucose even in the presence of oxygen. This species has orthologues of almost all *S. cerevisiae* genes that participate in fermentation and glycolysis, including 11 hexose transporters and the *SNF3*/*RGT2* transceptor genes involved in their regulation. Although the pathogenic *C. glabrata* presents many components of the cAMP-protein kinase A and glucose repression pathways, these regulatory systems have not been fully characterised.^9^ Importantly, while most yeast species in general assimilate and ferment several different sugar substrates (galactose, sucrose, maltose etc.), *C. glabrata* can only assimilate another sugar, the disaccharide trehalose (α-d-glucopyranosyl-(1-1)-α-d-glucopyranoside). This property was immediately recognised as a cost-effective and rapid means to identify this fungal pathogen,^10^ facilitating the therapy selection, especially in cases of candidaemia.^3^

While in the yeast *S. cerevisiae* two distinct pathways for trehalose assimilation have been described,^11^ we have shown that *C. glabrata* consumes and ferments trehalose, with similar parameters to those observed during glucose fermentation, due to the secretion of a highly active acid trehalase (EC 3.2.1.28) into the medium. Furthermore, cloning and heterologous expression of the acid trehalase gene (*CgATH1*) from *C. glabrata* allowed trehalose fermentation by *S. cerevisiae* cells.^12^ Indeed, the *CgATH1* acid trehalase was recently confirmed among the 119 proteins secreted by *C. glabrata* into the medium.^13^ Aiming to better characterise the function of the secreted acid trehalase, in the present report we have deleted the *CgATH1* gene from the *C. glabrata* genome and analysed the phenotypic consequences in the mutant strain, including the growth pattern when exposed to glucose or trehalose rich mediums, the trehalase activity, and its virulence in a murine model of infection, either in normal or immunocompromised mice. We also show that the majority of pathogenic/opportunistic *Candida* yeasts species are able to consume extracellular trehalose, due to the presence of genes encoding acid trehalases in their genomes.

## MATERIALS AND METHODS

*Yeast strains, media and growth conditions* - Unless otherwise stated, all chemicals used in this research were of analytical grade from Sigma-Aldrich (St. Louis, USA). The *C. glabrata* strains used in this study are described in the Supplementary data, Table I, and were maintained at -80⁰C. Standard rich YP medium (10 g L^-1^ Difco yeast extract, 20 g L^-1^ Difco Bacto peptone) or minimal YNB medium (6.7 g L^-1^ Difco Yeast nitrogen base) were used, supplemented with 20 g L^-1^ of glucose or trehalose or 30 g L^-1^ of glycerol. The pH of the rich medium was adjusted to pH 5.0 with HCl and, in the case of the minimal YNB medium, 50 mmol L^-1^ succinate-Tris pH 5.0 was used as buffer. Solid media contained 20 g L^-1^ Difco Bacto agar. Cells were grown aerobically at 28^o^C on a rotary shaker (160 rpm) in cotton-plugged Erlenmeyer flasks filled to 1/5 of the volume with medium and inoculated with 30-40 µg cell dry weight (CDW) mL^-1^. Alternatively, yeast cells were pre-grown overnight in 3 mL of YP-20 g L^-1^ glucose, and 1:100 dilutions of these pre-cultures were used to inoculate 100 μL of rich YP medium containing the indicated sugars in 96-well plates in a Tecan GENios microplate reader (Tecan, Männedorf, Switzerland), to determine the yeast growth at 30ºC. All wells in the plate were tightly sealed with AccuClear Sealing Film (E & K Scientific, Santa Clara, USA). The growth pattern of each culture was monitored by measuring the OD_600_ every 30 min, with high intensity orbital shaking between measurements. All growth experiments were repeated at least twice, observing that differences between strains were highly reproducible. To perform the murine experiments, the yeasts were subcultured in Sabouraud dextrose broth (Becton, Dickinson and Company, Sparks, USA) at 37⁰C for 24 h at 150 rpm before each assay.

*Strain construction* - Standard methods for DNA manipulation and analysis, as well as yeast transformation by the lithium acetate/single-stranded carrier DNA/polyethylene glycol method, were employed.^12^ The ORF of the *CgATH1* gene (*locus CAGL0K05137g*) on chromosome K of strain CBS138 (http://cbi.labri.fr/Genolevures/elt/GAGL) was disrupted by a PCR-based gene replacement procedure.^14^ Briefly, the *S. cerevisiae URA3* gene from plasmid YEp24 was amplified with primers PRODIGE-ATH1-F and PRODIGE-ATH1-R (Supplementary data, Table I) using Phusion^®^ High-Fidelity DNA Polymerase. The resulting PCR product of 924 bp, containing flanking regions (c. 60 nt.) of homology to the immediate upstream promoter and immediate downstream region of the *CgATH1* gene, was used to transform cells of the competent strain Bg14. After 2 h of cultivation on YP-20 g L^1^ glucose, the transformed cells were plated onto synthetic complete medium supplemented with 30 g L^1^ of glycerol and incubated at 28°C. Uracil-positive isolates were tested for proper genomic integration of the *ScURA3* gene at the *CgATH1 loci* by PCR using a set of three primers: VUATH1-F, VIATH1-R or VIURA3-R (Supplementary data, Table I).

*Determination of trehalase activity* - Trehalose hydrolysis by the periplasmic acid trehalase, and the activity in culture supernatants, was determined as previously described.^12^ Total trehalase activity in cell extracts was also determined using 100 mmol L^-1^ succinate-Hepes (pH 3.5 to pH 8.5), containing 2.5 mmol L^-1^ CaCl_2_ and 100 mmol L^-1^ trehalose. Enzyme activity is expressed as mU, where one unit corresponds to 1 µmol of glucose produced min^-1^ at 30^o^C. Glucose was determined by the glucose oxidase and peroxidase method using a commercial kit (BioDiagnostica-Laborclin, Pinhais, Brazil), and protein was quantified by the Bradford method.

*In vivo mice infections and treatments* - BALB/c isogenic male mice with 6-8 weeks (n = 5) were bred at the University of São Paulo (USP), Brazil, in an animal facility under pathogen-free conditions. Mice were kept in cages lined with wood shavings and closed with an autoclaved filter, and served (*ad libitum*) autoclaved food and water in order to maintain a sterile environment. Cages were changed twice a week in laminar flow hoods. The authors confirm that all procedures involving animals and their care were conducted according to the local ethics committee and international guidelines, approved by the Institutional Animal Care and Use Committee (protocol 042-127-02) of the Institute of Biomedical Sciences, USP.

Disseminated candidiasis in normal mice was induced by caudal intravenous (i.v.) inoculation of 3 × 10^5^ *C. glabrata* wild-type or *ath1*Δ yeast cells suspended in 100 μL of phosphate-buffered saline (PBS) on day 0. Animals were treated intraperitoneally (i.p.) with PBS (control) or fluconazole (20 mg/kg) every day for a week, starting on day 1. Alternatively, disseminated candidiasis in immunosuppressed animals was performed administrating two doses of 100 mg/kg cyclophosphamide i.p. four days and one day before infection, and again on day 3 and 7 post-infection, followed by i.v. infection with 1 × 10^3^ *C. glabrata* wild-type or *ath1*Δ yeast cells suspended in 100 μL of PBS on day 0. Animals were sacrificed eight days after infection, kidneys and spleen were removed and weighed and tissues were individually homogenised by mechanical disruption in 1 mL of PBS. Subsequently, 100 μL of these suspensions were diluted in 900 L of PBS and inoculated in plates containing brain-heart infusion (BHI) agar media (Becton Dickinson GmbH, Germany). Colonies were counted visually after 24 h of incubation at 37^o^C for fungal burden determination. Statistical analyses were performed using GraphPad Prism version 6.0 (GraphPad Software, San Diego, USA). Statistical comparisons were made by analysis of variance (one-way ANOVA) followed by a Tukey-Kramer post-test. p-values of < 0.05 indicated statistical significance. Results show mean ± standard deviation (SD) of three independent experiments.

*Phylogenetic analysis of trehalases and trehalose utilisation in pathogenic/opportunistic yeasts* - Phylogenetic trees were assembled with trehalases sequences from different pathogenic yeasts obtained from the Basic Local Alignment Search Tool (BLAST, https://blast.ncbi.nlm.nih.gov/Blast.cgi) or from the Yeast Gene Order Browser (YGOB) (http://ygob.ucd.ie) or Candida Gene Order Browser (*CGOB*) (http://cgob.ucd.ie) databases and then aligned using the Multiple Sequence Comparison by Log-Expectation tool (MUSCLE, https://www.ebi.ac.uk/Tools/msa/muscle). The alignment file was used to generate the phylogenetic tree using Akaike Information Criterion (AIC) in Smart Model Selection implemented in the PhyML environment (http://www.atgc-montpellier.fr/phyml-sms), with a bootstrap analysis of 1,000 replicates. For the management of the phylogenetic tree, we used the online tool Interactive Tree of Life (iTOL, https://itol.embl.de). Open reading frames from non-annotated sequences of putative trehalases were obtained through BLAST against Whole Genome Shotgun (WGS) or BioProject contigs in National Center for Biotechnology Information (NCBI), and then defined using ORFfinder tool (https://www.ncbi.nlm.nih.gov/orffinder/). Protein structure and functional domains of the amino acid sequences were analysed using the EMBL-EBI InterPro website (http://www.ebi.ac.uk/interpro/). The capacity of the different yeasts to assimilate (grow on) and/or ferment trehalose was verified in the Westerdijk Fungal Biodiversity Institute website (http://www.wi.knaw.nl/) using the description of type strains, as well as from a reference book.^15^

## RESULTS

**Fig. 1:**
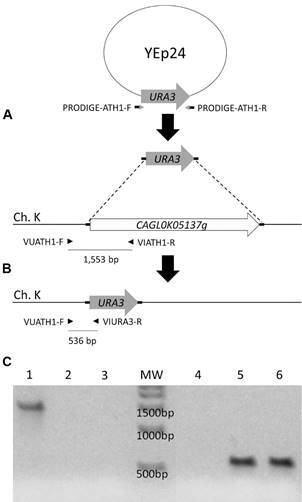
deletion of the *CgATH1* gene in *Candida glabrata* using the promoter-dependent disruption of genes (PRODIGE) method. (A) The *URA3* gene present in plasmid YEp24 was amplified with two primers having homology (~ 60 nt) to the immediately upstream and downstream regions of the *CgATH1* gene (ORF *CAGLOK05137g*) present in chromosome K. After transformation and selection in minimal YNB medium, the correct replacement of the *CgATH1* gene by *URA3* through recombination (B) was confirmed by PCR. (C) Gel electrophoresis of the products obtained with primers VUATH1-F and VIATH1-R and the genomic DNA of the wild-type strain (Lane 1) and its absence from two *aht1*Δ mutant strains (Lanes 2 and 3) or with primers VUATH1-F and VIURA3-R, which did not amplified any fragment from the wild-type strain (Lane 4), but produced the expected fragment from the two *aht1*Δ mutant strains) Lanes 5 and 6), indicating that the *ScURA3* gene was correctly integrated at the chromosome, deleting the *CgATH1* gene.

*Deletion of the CgATH1 gene impairs growth on trehalose by C. glabrata* - In chromosome K of the known genome of the *C. glabrata* strain CBS138 is located a gene (*locus CAGLOK05137g*) in synteny and with significant (68%) identity with the gene encoding the *ATH1* acid trehalase from *S. cerevisiae*.^16^ We have already shown that the expression of this *CgATH1* gene in *S. cerevisiae* allows efficient trehalose consumption and fermentation.^12^ In order to gain knowledge into the *in vivo* function of this gene in *C. glabrata*, we took advantage of the promoter-dependent disruption of genes (PRODIGE) method^14^ to delete this gene from the genome of strain Bg14 (Supplementary data, Table I). This method (Fig. 1) has been successfully used to analyse the effects of deleting specific genes in the susceptibility/resistance of *C. glabrata* strains challenged with different drugs, or even during macrophage killing.^17^ ^,^ ^18^

**Fig. 2:**
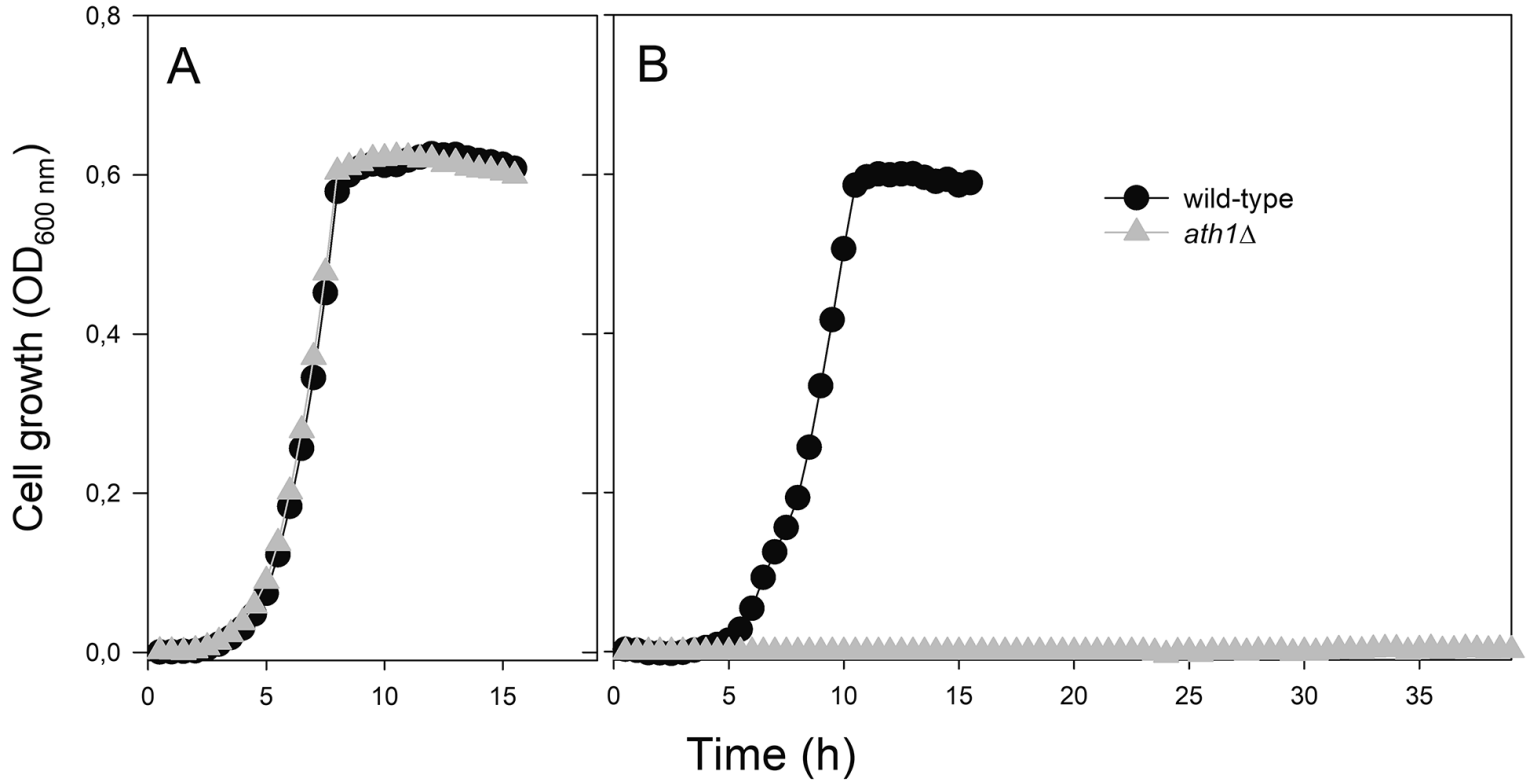
growth of the *Candida glabrata* wild-type or *aht1*Δ mutant strain in rich YP medium containing 20 g L^-1^ of glucose (A) or trehalose (B) as carbon source. Yeast growth at 30ºC was monitored with a Tecan GENios microplate reader by measuring the OD_600_ every 30 min, with high intensity orbital shaking between measurements.

Since we have previously shown that the acid trehalase is glucose repressed, and high levels of secreted enzyme activity were obtained when the yeast cells are grown in glycerol,^12^ the transformants were selected in minimal YNB medium plates with 30 g L^-1^ of glycerol as carbon source. From more than 20 colonies that showed growth on YNB-30 g L^-1^ glycerol plates, two of them failed to grow in YNB-20 g L^-1^ trehalose plates and PCR analysis confirmed that *ScURA3* gene was correctly integrated in chromosome K, deleting the *CgATH1* gene in these strains (Fig. 1). One of these *aht1*Δ mutant strains was used for further characterisation, and as can be seen in Fig. 2, this *aht1*Δ strain could grow normally in rich YP-20 g L^-1^ glucose medium (Fig. 2A), but failed to grow in rich medium containing 20 g L^-1^ trehalose as carbon source (Fig. 2B). Same results were obtained in synthetic YNB medium with 20 g L^-1^ of glucose or trehalose (data not shown).

*Trehalase activity in C. glabrata cells* - The wild-type strain had a periplasmic acid trehalase activity of 58.3 ± 14.2 mU/mg CDW after growth on rich YP medium with 20 g L^-1^ of glucose, and an activity of 131 ± 48 mU/mg CDW when grown on the same medium containing 30 g L^-1^ of glycerol. The *aht1*Δ mutant strain showed less than 0.3 mU/mg CDW of periplasmic trehalase activity after growth on any of these carbon sources. The wild-type strain secreted more than 480 ± 130 mU/mL of acid trehalase activity into the medium after growth on glucose or glycerol, but we could not detect any acid trehalase activity secreted into the medium by the *aht1*Δ mutant strain.

**Fig. 3:**
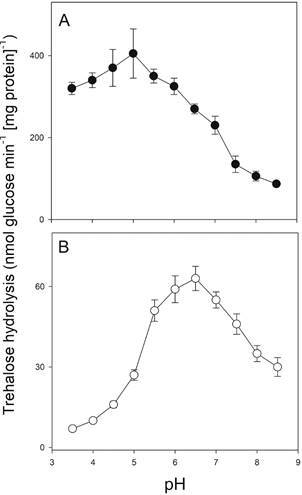
trehalase activity in cell extracts of the *Candida glabrata* wild-type (A) or *aht1*Δ mutant strain (B) determined in the indicated pH after growth of the cells for 24 h on rich YP medium containing 20 g L^-1^ of glucose.

Besides the *CgATH1* gene encoding for an extracellular acid trehalase, the known genome of *C. glabrata* contains two other putative trehalase genes (*CAGLOC04323g* and *CAGLOM10439g*) that are syntenic with a couple of paralogs with 70% identity that arose from the WGD event, and share 67-79% identity with the *NTH1* and *NTH2* genes from *S. cerevisiae*, which encode for the intracellular neutral trehalases.^16^ However, due to the very high activity of the acid trehalase present in the wild-type *C. glabrata* strain, it was difficult to visualise the activity of the cytoplasmic neutral trehalase in cell extracts (Fig. 3A). However, this activity can be clearly seen in cell extracts of the *aht1*Δ strain (Fig. 3B), with an optimal pH of 6.5-7.0. It is important to note that the neutral trehalase activity in *aht1*Δ yeast cells was very low, when compared to the high acidic trehalase activity present in the wild-type strain. No differences were observed in the intracellular trehalose concentration or intracellular trehalose mobilisation during growth on glucose by the wild-type or *aht1*Δ yeast strains (data not shown).

**Fig. 4:**
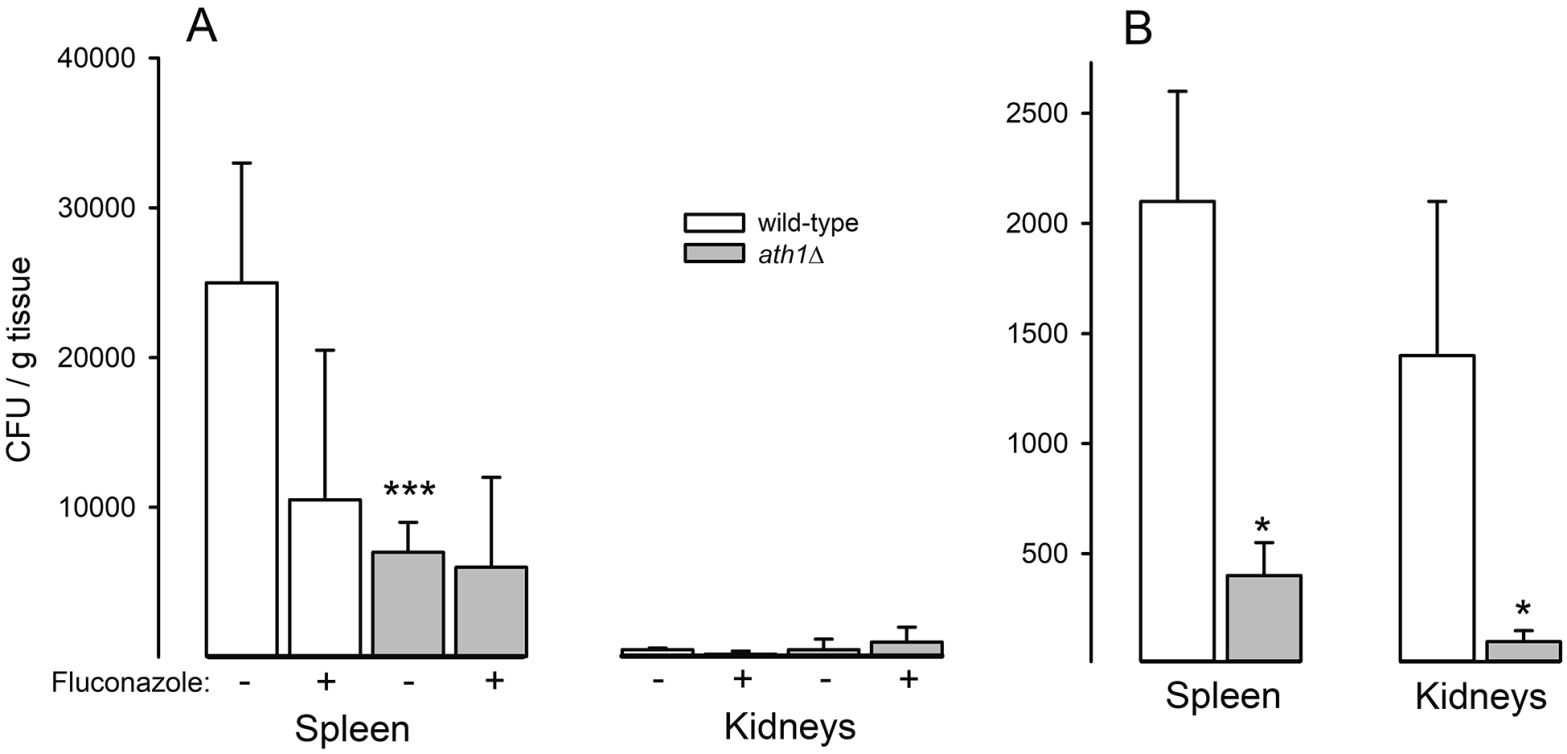
fungal burden in spleens and kidneys from (A) immunocompetent BALB/c mice inoculated with 3 x 10^5^ yeast cells of *Candida glabrata* wild-type or *ath1*Δ strain and treated daily with (or without) fluconazole as indicated. (B) Cyclophosphamide-treated immunosuppressed mice inoculated with 1 × 10^3^ yeast cells of *C. glabrata* wild-type or *ath1*Δ strain. In all cases, colony-forming units (CFUs) were determined eight days post-challenge. Statistical analysis was carried out using the analysis of variance (one-way ANOVA) with post Tukey-Kramer test: * p < 0.01; *** p < 0.0001 between the wild-type and *ath1*Δ strain.

*The acid trehalase encoded by CgATH1 is involved in C. glabrata virulence* - The effect of deleting *CgATH1* on the virulence of *C. glabrata* was determined in a murine model of disseminated infection, both in normal or immunocompromised mice (Fig. 4). After caudal i.v. yeast inoculation in immunocompetent mice, a high number of colony-forming units (CFUs) of the *C. glabrata* wild-type strain were found in the mice spleen eight days after infection, but much less in the kidney (Fig. 4A). The *aht1*Δ mutant strain underwent a significant loss of virulence, as shown by the lower number of CFUs found in the spleen, compared to those recorded by the wild-type strain, reaching the same low values observed when the infected mice were treated with fluconazole (Fig. 4A). Actually, treatment with fluconazole did not further reduce the fungal burden of the *aht1*Δ strain. A slightly different pattern was observed in immunocompromised mice since besides the spleen, kidneys were also significantly infected (Fig. 4B), but in both tissues the fungal burden by the *aht1*Δ mutant strain was significantly lower than the *C. glabrata* wild-type strain. Thus, our results indicate that extracellular trehalose hydrolysis and metabolisation are important for virulence of this fungal pathogen in the mammalian host. Considering that extracellular trehalases are known virulence factors for entomopathogenic fungus, since trehalose is the main sugar in the haemolymph of insects, we refrained from using an alternative insect infection bioassay (e.g., with *Galleria mellonella* larvae or silkworm) to determine the virulence of the *ath1*Δ mutant strain.

**Fig. 5:**
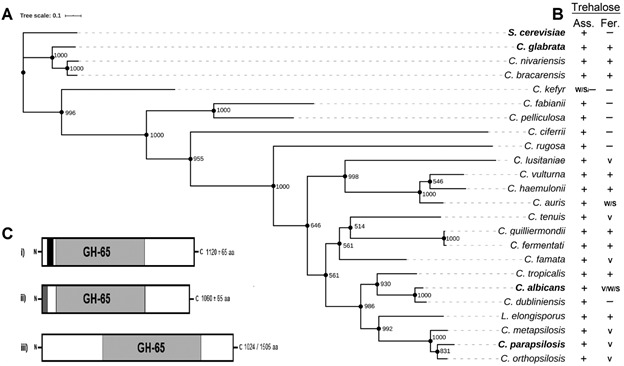
acid trehalases and trehalose assimilation and fermentation by pathogenic/opportunistic *Candida* species. (A) Phylogenetic tree of characterised (bold) and other predicted acid trehalases present in the indicated yeast species. The numbers next to the nodes represent the bootstraps values (based on 1,000 replications). (B) Data regarding extracellular trehalose assimilation (Ass.) or fermentation (Fer.) by the yeasts - +: positive; −: negative; w: weak; s: slow/delayed; v: variable. (C) Schematic representation of the acid trehalases and their functional domain content, including the glycosyl hydrolase family 65 (GH-65) trehalase domain (grey), the TM segment (black, i), a signal peptide (dark grey, ii) or even absence of these last two domains in some predicted enzymes (iii).

*Trehalases and extracellular trehalose utilisation by pathogenic/opportunistic Candida yeasts* - We analysed the ability to assimilate (grow on) and ferment the disaccharide trehalose by the major pathogenic/opportunistic *Candida* yeast species^1^ with assembled genome sequences available (a total of 28 species, including the opportunistic yeast *S. cerevisiae*) and performed a phylogenetic analysis of the genes encoding trehalases present in those yeasts (Fig. 5). A total of 24 *Candida* yeast species had genes encoding for acid trehalases (with ≥ 35% identity with *ATH1* from *S. cerevisiae* (Supplementary data, Tables II, III) and all of them were able to assimilate trehalose from the medium (including “weak” or “slow/delay” phenotypes (Fig. 5A, B)*.* More than 70% of these trehalose positive species also showed some degree of trehalose fermentation (including “positive”, “variable”, “slow/delay” or “weak” phenotypes).

Considering the five major pathogenic *Candida* yeast species (*C. albicans*, *C. glabrata*, *C. krusei*, *C. parapsilosis* and *C. tropicalis*), responsible for approximately 93% of all invasive candidiasis cases,^1^ only *C. krusei* (syn. *Pichia kudriavzevii*) lacks a gene that encodes for an acid trehalase, and is unable to assimilate (or ferment) trehalose. Regarding the other 23 emerging/opportunistic pathogenic yeast species (all with less than 1% of individual incidence), 20 of them had genes encoding acid trehalases and showed growth on trehalose, of which 13 also had some degree of trehalose fermentation (Fig. 5B). We could not find genes encoding for acid trehalases in three other emerging/opportunistic *Candida* yeast species: *C. inconspicua* (syn. *Pichia cactophila*), *C. lipolytica* (syn. *Yarrowia lipolytica*) or *C. norvegensis* (syn. *P. norvegensis*), and as expected none of them could assimilate or ferment trehalose. It is important to note that the three pathogenic yeasts within the *Nakaseomyces* genus (*C. glabrata*, *C. bracarensis* and *C. nivariensis*)^5^ have acid trehalases and assimilate and ferment trehalose (Fig. 5), while the three non-pathogenic species (*C. castellii*, *N. bacillisporus* and *N. delphensis*) do not consume or ferment this sugar. Overall, our analysis reveals that more than 85% of the major pathogenic and emerging/opportunistic *Candida* species have genes encoding for an acid trehalase and show extracellular trehalose assimilation, or even fermentation.

The acid trehalase encoded by the *CgATH1* gene belongs to the glycosyl hydrolase family 65 (GH-65) of the Carbohydrate-Active enZyme database (CAZy, http://afmb.cnrs-mrs.fr/CAZY). These enzymes are extracellular because they could present a transmembrane domain close to the N-terminal (TM) (Fig. 5C-i) responsible for anchoring the protein at the plasma membrane and/or cell wall ^(^ ^19^ or even promoting the secretion of the enzyme into the medium, as in the case of *C. glabrata*.^12^ Only five yeast species (*C. auris*, *C. ciferrii*, *C. fermentati*, *C. kefyr* and *C. pelliculosa*) have slightly shorter proteins with a signal peptide at the N-terminal end of the sequence (Fig. 5C-ii), indicating that they are also probably secreted into the medium. Only two species (*C. haemulonii* and *C. tenuis*) have proteins with N-terminal domains with different sizes and lacking any of these targeting signals (Fig. 5C-iii). Thus, their involvement in extracellular trehalose hydrolysis remains to be proven experimentally. It is important to note that trehalose might also be directly transported into the cell, where it can be hydrolysed by cytoplasmic neutral trehalases, as has been described in *S. cerevisiae*.^11^ An initial survey of putative trehalose transporters in *C. haemulonii* and *C. tenuis* revealed several genes (data not shown) with all the characteristics described for such permeases, including those amino acids required for sugar binding and transport.^20^

Indeed, we found genes encoding for neutral trehalases in all the pathogenic yeast species analysed. Supplementary data, Figure, shows the enzymes found in the 24 pathogenic yeasts of Fig. 5 plus two other yeast species that lack acid trehalase genes (*C. krusei* and *C. incons-picua*). Neutral trehalases belong to the GH family 37 (GH-37) (which includes a Ca^2+^ binding domain) in the CAZy database and, as mentioned before, these enzymes are responsible for intracellular hydrolysis of trehalose.^16^ In *S. cerevisiae* and other yeasts that underwent the WGD, two paralogs are found: *NTH1* and *NTH2*, but most pathogenic/opportunistic yeast species had genes encoding for a single neutral trehalase, either with higher identity to *NTH1* (21 genes) or to *NTH2* (seven genes). Surprisingly, *C. krusei* been the more distantly related species, also had two genes encoding for neutral trehalases (Supplementary data, Figure, Table II).

## DISCUSSION

The disaccharide trehalose is an important sugar for several microorganisms, plants and insects. Trehalose is considered not only a storage carbohydrate and stress-protectant, but it also acts as regulator of many cellular processes. Since humans do not synthetise trehalose, the enzymes involved in trehalose metabolism by pathogenic yeasts have been studied as interesting and potentially antifungal targets for chemotherapy.^21^ Our results show that the *C. glabrata* secreted acid trehalase encoded by the *CgATH1* gene is required for growth on trehalose and contributes to the virulence of the yeast. There are at least two other examples were deletion of the acid trehalase decreases infectivity by pathogenic yeasts. The acid trehalase present in *C. albicans*, which is required for growth on trehalose,^22^ is involved in the virulence of this major pathogenic yeast.^23^ Same results have been reported for the acid trehalase of *C. parapsilosis*.^24^ More recently, a trehalase required for growth on trehalose has been also implicated in virulence of the bacteria *Burkholderia pseudomallei*.^25^

An intriguing issue raised by these results is why these pathogenic microorganisms have the ability to consume extracellular trehalose as carbon source (Fig. 5), since this sugar is not normally found in the environment or mammalian host, and its implications for virulence. A possible scenario is that during colonisation and infection, when many microorganisms are being killed by the harsh environment and immune defences of the host (thus, liberating trehalose into the medium), having the ability to consume (or even ferment) trehalose could be an advantageous trait for a pathogen. Indeed, humans also expresses the enzyme trehalase as a glycoprotein in the small intestine and renal brush-border membranes, and while the trehalase in the intestine is involved in the hydrolysis of ingested trehalose, in turn, the physiological role of trehalase in the kidney has remained more elusive, but certainly could hydrolyse trehalose released by dying microorganisms present in the urinary tract. Dietary trehalose is also a concern, since recent results indicate that hypervirulent epidemic *Clostridioides difficile* ribotypes have enhanced capacity to consume trehalose, and this sugar increases the virulence of such strains in a murine model of infection.^26^ Unfortunately, the known trehalase competitive inhibitor validamycin A had limited antifungal effects with *C. albicans*.^27^ However, a recent publication showed that validamycin A had a synergistic inhibitory effect with amphotericin B against the pathogenic fungus *Aspergillus flavus*, without cytotoxic effects in human bronchial epithelial cells, indicating its possible use *in vivo* for treatment of fungal infections.^28^ Nevertheless, new trehalose analogues, including those designed to resist enzymatic degradation that inhibit trehalose utilisation by hypervirulent *C. difficile* ^29^ may constitute new clinically interesting antifungal-related compounds. Thus, more studies are required to elucidate the role of extracellular trehalose and the high levels of secreted acid trehalase has in the physiopathology of *C. glabrata* and other yeast pathogens (Fig. 5).

Our analysis of both experimentally characterised and predicted trehalases in the major pathogenic/opportunistic *Candida* yeast species (Fig. 5 and **Supplementary data**, Figure) revealed that acid trehalases have the classical GH-65 domain and either a TM (70% of the acid trehalases) (Fig. 5C-i) or signal peptide (21%) (Fig. 5C-ii). These sequences allow extracellular localisation, or even secretion, of the enzyme into the medium, as already described for known acid trehalases of yeast and other fungi.^19^ The only exceptions were the predicted acid trehalases found in *C. haemulonii* and *C. tenuis*, lacking any of these targeting domains (Fig. 5C-iii), and thus the mode of extracellular trehalose assimilation by these yeasts needs further studies. Regarding the neutral trehalases, we found a more diverse scenario regarding this enzyme in the analysed pathogenic/opportunistic yeast, with the majority of species having a single enzyme with the GH-37 domain (either of the *NHT1* or *NHT2* type of neutral trehalases), while some yeast species that underwent the WGD had both paralogs (A and B-i in **Supplementary data**, Figure). Indeed, within the WGD yeast species, some have a single gene homologous to *NTH1* (e.g., *Kazachstania naganishii*), others a single gene homologous to *NTH2* (e.g., *K. africana*, *Naumovozyma castellii* and *N. diarenensis*), while other yeast species have genes homologous to the two neutral trehalases (e.g., *Tetrapisispora blattae*, *T. phaffii* and *Vanderwaltozyma polyspora*).

It is also significant to note that the intracellular neutral trehalase activity in *C. glabrata* is only clearly detected in the *ath1*Δ mutant strain (Fig. 3), and since this enzyme in *S. cerevisiae* is a known target of the cAMP/PKA nutrient signalling pathway, promoting the switch between respirative/gluconeogenic growth and fermentative growth,^30^ the mutant *ath1*Δ strain is a promising platform to characterise the *C. glabrata* orthologues of the known receptors and transceptors (transporter receptors) involved in nutrient sensing and signalling through the cAMP/PKA signalling pathway. In fungal pathogens, the sensing of sugars is important for a number of virulence attributes, including adhesion, oxidative stress resistance, biofilm formation, morphogenesis, invasion and antifungal drug tolerance, thus the understanding of sugar sensing and metabolism may offer new valuable antifungal drug targets.^9^

In conclusion, in *C. glabrata*, the deletion of the *CgATH1* gene encoding for the secreted acid trehalase impairs the use of this carbon source for growth, reveals the activity of the intracellular neutral trehalase, and reduces the fungal burden in a murine model of infection, both in normal or immunocompromised mice.
