# Supplementary material for: The secreted acid trehalase encoded by the CgATH1 gene is involved in Candida glabrata virulence
Source: Mem Inst Oswaldo Cruz. 2020 Oct 30;115:e200401. doi: 10.1590/0074-02760200401 (PMC7607559; doi:10.1590/0074-02760200401)
Supplement: Supplementary file 1 [file 1678-8060-mioc-115-e200401-s.pdf]

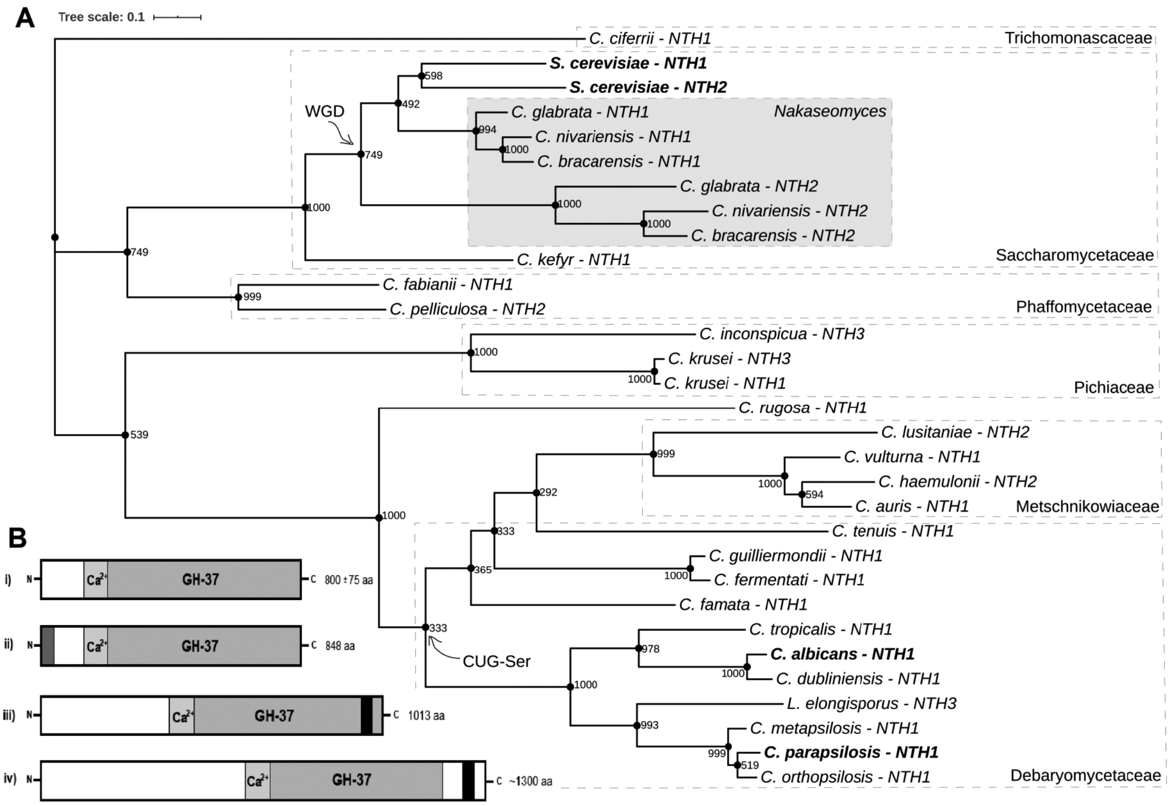

Neutral trehalases in pathogenic/opportunistic *Candida* species. (A) Phylogenetic tree of characterised (bold) and predicted neutral trehalases present in the indicated yeast species. The numbers next to the nodes represent the bootstraps values (based on 1,000 replications). Dashed lines delimit the species within six families of the order Saccharomycetales and the gray rectangle corresponds to *Nakaseomyces* clade. The common ancestor of yeasts that underwent the WGD event and of those from the CUG-Ser clade are pointed by curved arrows. (B) A schematic representation of the neutral trehalases and their functional domain content, including the GH-37 (and Ca^{2+} binding domain) trehalase signature (grey and light grey, respectively) found in all neutral trehalases (i), with the exception of *C. vulturna* that had a predicted enzyme with a signal peptide at the N-terminal end of the protein (dark grey, ii) and in the case of *C. krusei* and *L. elongisporus* that had a TM segment (black, iii and iv) at the C-terminal domain in these bigger neutral trehalases. The yeast *C. inconspicua* also harbours a bigger neutral trehalase (iv), but lacks the C-terminal TM segment. We have called these bigger and different neutral trehalases "*NTH3*" (Supplementary data, Table II) to distinguish them from *NTH1*/*NTH2*.

TABLE I

Candida glabrata strains and oligonucleotides used in this study

Yeast strain or primer	Relevant genotype or description	Source or reference
Yeast strains		
Bg2	Wild-type clinical isolate	Fidel et al.[31]
Bg14	*ura3*Δ::*Tn903 NeoR* derivative of Bg2	Cormack & Falkow[32]
*ath1*Δ	*ath1*Δ::*ScURA3* derivative of Bg14	This study
Primers		
PRODIGE-ATH1-F	AACTCTGTAAATAATAACAAGACAACACTGCAAGTAGCGAGGTCT<br>GGAAAGAGAAGTACAATGTCGAAAGCTACATATAAGG	Invitrogen
PRODIGE-ATH1-R	TTACTCTGCCAAGTAATTATGTGTAGATATGAGCTAGATCCCCAGT<br>TTCATTCTTGAAGGTTAGTTTTGCTGGCCGCATC	Invitrogen
VUATH1-F	GATTGCTACAGCATATCTATCC	Invitrogen
VIATH1-R	TGTCATAGATAGTTACCGGT	Invitrogen
VIURA3-R	CAGCAACAGGACTAGGATGAG	Invitrogen

cc BY

TABLE II

Information regarding trehalase genes/proteins found in pathogenic/opportunistic *Candida* yeast species

Yeast specie	Teleomorph/synonym	GenBank/contig/BioProject #, number of amino acids and (% identity with *S. cerevisiae ATH1*, *NHT1* or *NTH2*)			
		ATH1	*NTH1*	*NTH2*	*NTH3*[a]
S. cerevisiae	–	NP_015351.1<br>1,211 (100)	NP_010284.1<br>751 (100)	NP_009555.1<br>780 (100)	–
C. glabrata	*Torulopsis glabrata*	CAG61411.1<br>1,212 (67)	XP_449801.1<br>765 (79)	KTA98647.1<br>750 (67)	–
C. bracarensis	–	CAPU01000173.1[b]<br>1,215 (67)	CAPU01000123.1[b]<br>768 (77)	CAPU01000056.1[b]<br>750 (67)	–
C. nivariensis	–	CAPV01000115.1[b]<br>1,217 (67)	CAPV01000093.1[b]<br>769 (77)	CAPV01000109.1[b]<br>736 (70)	–
C. auris	–	XP_028890702.1<br>1,082 (38)	XP_028893077.1<br>(57)	–	–
C. albicans	–	RLP61977.1<br>1,078 (39)	EEQ43188.1<br>(55)	–	–
C. ciferrii	*Trichomonascus ciferrii*	KAA8916057.1<br>1,011 (38)	KAA8915314.1<br>665 (61)	–	–
C. dubliniensis	*C. dublinionensis*	XP_002417309.1<br>1,078 (39)	XP_002421663.1<br>902 (55)	–	–
C. fabianii	*Cyberlindnera fabianii/ Hansenula fabianii*	ONH69371.1<br>1,059 (46)	ONH67346.1<br>755 (65)	–	–
C. famata	*Debaryomyces hansenii*	XP_459109.2<br>1,100 (40)	XP_457082.2<br>846 (58)	–	–
C. fermentati	*Meyerozyma caribbica*	BADS01000007.1[b]<br>1,056 (39)	OCTK01000006.1[b]<br>685 (58)	–	–
C. guilliermondii	*M. guilliermondii*	EDK36433.2<br>1,149 (38)	EDK38463.2<br>821 (56)	–	–
C. haemulonii	*C. haemulonis*	XP_025340541.1<br>1,505 (38)	–	XP_025339566.1<br>815 (56)	–
C. inconspicua	*Pichia cactophila/ Torulopsis inconspicua*	–	–	–	TID24615.1<br>1,294 (56)
C. kefyr	*Kluyveromyces marxianus*	BAP73405.1<br>1,157 (54)	XP_022677201.1<br>753 (71)	–	–
C. krusei	*P. kudriavzevii*	–	PPIQ02001010.1[b]<br>984 (56)	–	MQVM01000062.1[b]<br>1,304 (56)
C. lusitaniae	*Clavispora lusitaniae*	QFZ26320.1<br>1,190 (39)	–	QFZ29246.1<br>767 (57)	–
C. metapsilosis	*C. parapsilosis group III*	PQNC01000002.1[b]<br>1,045 (38)	CBZN020000090.1[b]<br>895 (56)	–	–
C. orthopsilosis	*C. parapsilosis group II*	XP_003866649.1<br>1,070 (38)	XP_003866206.1<br>893 (55)	–	–
C. parapsilosis	*Monilia parapsilosis*	CCE43253.1<br>1,071 (38)	CCE41826.1<br>903 (56)	–	–
C. pelliculosa	*Wickerhamomyces anomalus*	XP_019041443.1<br>992 (47)	–	XP_019037428.1<br>763 (65)	–
C. tenuis	*Yamadazyma tenuis*	XP_006686328.1<br>1,024 (40)	XP_006689954.1<br>828 (55)	–	–
C. tropicalis	*Oidium tropicale*	XP_002549896.1<br>1,083 (39)	XP_002546238.1<br>884 (57)	–	–
C. rugosa	*Diutina rugosa*	KAA8906963.1<br>1,070 (35)	KAA8899321.1<br>827 (57)	–	–
C. vulturna	–	PRJNA560499[c]<br>1,087 (38)	PRJNA560499[c]<br>848 (57)	–	–
Lodderomyces elongisporus	*S. elongisporus*	XP_001528290.1<br>1,140 (38)	–	–	XP_001527945.1<br>1,013 (55)

a: percentages of *NTH3* identities are referred to *S. cerevisiae NTH1*; *b*: these accession numbers correspond to contigs from Whole Genome Shotgun (WGS) contigs where sequences of putative trehalases were found (the analysed sequences can be found between the limits presented in Supplementary data, Table III; *c*: this is the BioProject/NCBI number of *C. vulturna* genome[33] (the analysed sequences from contigs 1 (*ATH1*) and 3 (*NTH1*) can be found between the limits presented in Supplementary data, Table III).

TABLE III
Limits in Whole Genome Shotgun (WGS) or BioProject contigs where the putative trehalases were found

	Trehalase			
Yeast specie	*ATH1*	*NTH1*	*NTH2*	*NTH3*
C. bracarensis	24540 to 28184	9003 to 11306	149913 to 152162	–
C. nivariensis	276981 to 280631	102899 to 105205	258267 to 260474	–
C. fermentati	921450 to 924617	68151 to 70205	–	–
C. krusei	–	5640 to 8618	–	4792 to 7725
C. metapsilosis	1007111 to 1010245	370091 to 372775	–	–
C. vulturna	1657999 to 1661259	1876169 to 1878712	–	–
